# Trait Impulsivity Is Independent of Mild Cognitive Impairment in a Parkinson's Disease Cohort

**DOI:** 10.1155/2019/2672075

**Published:** 2019-10-01

**Authors:** Ashani Jeyadevan, Megan C. Bakeberg, Michelle Byrnes, Jade Kenna, Soumya Ghosh, Rick Stell, Sue Walters, Tess Evans, Sarah McGregor, Malcolm Horne, Frank L. Mastaglia, Ryan S. Anderton

**Affiliations:** ^1^School of Health Sciences, University of Notre Dame Australia, Fremantle, WA, Australia; ^2^Perron Institute for Neurological and Translational Science, Nedlands, WA, Australia; ^3^Centre for Neuromuscular and Neurological Disorders, University of Western Australia, Nedlands, WA, Australia; ^4^Institute of Health Research, University of Notre Dame Australia, Fremantle, WA, Australia; ^5^Florey Institute for Neuroscience and Mental Health, University of Melbourne, Parkville, Victoria 3010, Australia; ^6^Centre for Clinical Neurosciences and Neurological Research, St. Vincent's Hospital Melbourne, Fitzroy, Victoria 3065, Australia

## Abstract

**Introduction:**

Patients with Parkinson's disease (PD) commonly experience cognitive deficits and some also develop impulse control disorders (ICDs); however, the relationship between impulsivity and cognitive dysfunction remains unclear. This study investigated whether trait impulsivity associates with mild cognitive impairment (MCI), or is altered in a PD patient cohort with MCI.

**Methods:**

A total of 302 patients with idiopathic PD were recruited sequentially from three Australian Movement Disorder clinics. Based on cognitive scores, participants were divided into two groups, one defined as having mild cognitive impairment (PD-MCI; *n* = 113) and the other with normal cognitive function (PD-C; *n* = 189). Trait impulsivity was evaluated using the Barrett Impulsiveness Scale 11 (BIS-11). Total impulsivity scores, as well as subscale scores, were compared between PD-C and PD-MCI groups.

**Results:**

The PD-MCI cohort had significantly lower scores in all cognitive domains, and mirrored expected clinical differences in medication, motor symptoms, and disease duration, when compared to the PD-C cohort. Self-reported impulsivity was not significantly different between groups, nor was there a difference within first-order subscale scores: attention (*p*=0.137), cognitive instability (*p*=0.787), self-control (*p*=0.503), cognitive complexity (*p*=0.157), motor impulsivity (*p*=0.559), or perseverance (*p*=0.734) between the PD-MCI and PD-C groups.

**Conclusions:**

These findings suggest that impulsive traits and behaviors are independent of changes in cognitive state and are not altered in PD patients with mild cognitive impairment.

## 1. Introduction

Parkinson's disease (PD) is a complex neurodegenerative disorder in which the cardinal motor symptoms are accompanied by a variety of nonmotor symptoms (NMS) including olfactory, autonomic, psychiatric, and cognitive dysfunction. Prodromal cognitive deficits are of particular interest, offering potential insight into disease progression and early diagnosis, as well as targets for disease-modifying therapies. Cross-sectional studies have observed that approximately 30% of people with PD (PwP) have dementia [1, 2]. Furthermore, a 20-year longitudinal Australian study revealed that over 80% of PwP develop increasing cognitive impairment as the disease progresses and eventually become demented [3].

There has also been increasing awareness of the occurrence of abnormal impulsive-compulsive behaviors in PD patients as the disease progresses, particularly those treated with dopamine agonist drugs, which have been found to occur in as many as ∼46% of patients followed over a 5-year period [4–6]. These have been collectively termed impulse control disorders (ICDs), include pathological gambling, shopping, eating, hoarding, and hypersexuality, as well as compulsive use of dopaminergic medications (“dopamine dysregulation syndrome”) [7, 8], and are all regarded as compulsive reward-seeking forms of behavior. They confer heightened levels of distress for both patients and carers, as well as having serious implications for quality of life [9]. Heightened trait impulsivity is present in a subset of PD patients, particularly males [10], and is considered a risk factor for the development of ICDs.

The neural substrate of ICDs and increased impulsivity in PD is thought to involve dysregulation in mesolimbic and mesocortical networks, and changes in dopamine receptor (D2 and D3) binding in the ventral striatum [5, 11], but specific neuropathological correlates have not been identified. However, based on the disease staging studies by Braak et al., it might be envisaged that changes in impulsivity could correlate with Stage V, in which the Lewy pathology extends to the mesolimbic cortex, and could precede, or overlap with, the development of impaired cognition, as there is further extension to neocortical areas in Stage VI of the disease [12].

Although impulsivity and cognitive impairment frequently coexist in PD, it is unclear whether there are any interactive effects between them during the course of the disease. A link between impulsive traits and low cognitive scores has been observed in other cohorts, such as children with attention-deficit hyperactive disorder (ADHD) [13]. In addition, preliminary evidence suggests that, in PD, cognitive characteristics such as poor executive abilities, as well as poor action control and response inhibition, and certain personality traits such as negative affectivity and high premorbid levels of novelty seeking, may have impact on impulsivity and be risk factors for the development of ICDs [6, 11, 14]. However, relatively little is known about the link between cognitive abilities and behavioral changes in patients with PD, and there have been conflicting findings in the literature [15–17]. In particular, the relationship between subclinical impulsiveness and mild cognitive impairment (PD-MCI) in patients without a diagnosed ICD remains unclear, as previous studies have focused on cohorts with diagnosed behavioral disorders.

Accordingly, the aim of this cross-sectional study was to evaluate the relationship between MCI and subclinical impulsivity as measured using the Barratt Impulsiveness Scale 11 (BIS-11) in an Australian multicenter PD patient cohort. More specifically, we questioned whether in PD patients with MCI, there are changes in attentional and motor impulsiveness, and in measures of cognitive complexity and instability.

## 2. Methodology

### 2.1. Participants

A total of 302 patients were recruited sequentially into the Australian Parkinson's Disease Registry (APDR) from Movement Disorder Clinics at the Perron Institute in Perth, St. Vincent's Hospital in Melbourne, and Royal North Shore Hospital in Sydney. In all cases, the diagnosis of idiopathic PD was confirmed in accordance with the UK Brain Bank criteria prior to inclusion in the study. At the time of all assessments, patient response to medication was at optimum levels (“ON” period). Written informed consent was obtained from all participants, in accordance with the National Health and Medical Research Council guidelines. Patients who were unable to complete the cognitive and impulsivity protocols or had a diagnosed ICD were excluded from this study.

### 2.2. Demographic and Clinical Assessment

Patient demographic and clinical information was collected, including age, gender, date of diagnosis, motor symptom severity, smoking status, dopaminergic medications, and deep brain stimulation (DBS) history. For each patient, the total daily intake of all dopaminergic medications was converted to a levodopa equivalent dose (LED), as described elsewhere [18]. Part III of the Movement Disorders Society Unified Parkinson's Disease Rating Scale (MDS-UPDRS III) was employed to evaluate the severity of motor symptoms and was conducted by a clinician or trained research nurse. Lastly, patient quality of life data were collected using the self-assessed, 39-item Parkinson's Disease Questionnaire (PDQ-39) [19].

### 2.3. Assessment of Impulsivity

The Barratt Impulsiveness Scale 11 (BIS-11) is a self-report validated evaluation and assesses impulsivity as a multifaceted entity [20]. Second-order attentional impulsiveness is described as an inability to concentrate and can be categorised into first-order subscales for attention and cognitive instability. Motor impulsiveness is the tendency to act without thinking, with first-order motor and perseverance scales. Lastly, nonplanning impulsiveness is an inability to plan for the future, within which there are first-order subscales for cognitive complexity and self-control. The BIS-11 is entirely self-rated, with each item marked on a four-point scale, giving patients a score between 30 and 120. As the scoring scheme is reversed in some questions, each question was marked individually to ensure that higher BIS-11 scores gave a true indication of heightened impulsiveness.

### 2.4. Assessment of Global Cognition

Global cognition impairments were assessed using Addenbrooke's Cognitive Examination-Revised (ACE-R) [21], which has been used previously to determine cognitive impairment in PD patient cohorts [22, 23]. The ACE-R evaluated five prominent cognitive domains, with a maximum total score of 100 points: orientation and attention (18 points), memory (26 points), verbal fluency (14 points), language (26 points), and visuospatial (16 points) abilities. In all cognitive domains, lower scores represent poorer cognitive abilities. On the basis of the ACE-R scores, patients were allocated to either an MCI group (PD-MCI) or a cognitively normal group (PD-C), according to a verified total ACE-R score cut-off of 88.5 (sensitivity 0.68 and specificity 0.91) [24].

### 2.5. Statistical Analysis

All data were analysed using IBM-SPSS (v. 25, IBM Corporation) and presented as mean ± standard deviation unless otherwise stated. For comparisons between PD-C and MCI groups, nonparametric Mann–Whitney *U* test was performed. Models were corrected for covariates that were demonstrated to significantly differ between PD-C and PD-MCI cohorts. A significant nominal *p* value of <0.05 was employed.

## 3. Results

### 3.1. Cognitive Characteristics of PD-C and PD-MCI Groups

The cohort was initially divided into two groups based on the presence or absence of mild cognitive impairments (PD-MCI and PD-C, respectively), as determined by ACE-R scores. ACE-R subdomains were compared between cognitive groups, revealing significant differences in each subdomain. Throughout attention and orientation (*p* < 0.001), memory (*p* < 0.001), fluency (*p* < 0.001), language (*p* < 0.001), and visuospatial (*p* < 0.001) domains, significant differences were present between the PD-C and PD-MCI subgroups (Table 1).

### 3.2. Cohort Demographics and Clinical Differences between PD-C and PD-MCI Cohorts

Table 2 summarizes clinical and demographic characteristics of the complete patient cohort (*n* = 302), the PD-MCI subgroup, and the PD-C subgroup. Patients in the PD-MCI group were more likely to be older (63.2 ± 9.48 vs. 66.4 ± 8.51, *p*=0.003), have a longer disease duration (7.39 ± 5.09 vs. 9.75 ± 6.10, *p* < 0.001), be male (OR = 4.24, *p*=0.039), to have more severe motor symptoms (17.7 ± 11.0 vs. 23.6 ± 15.4, *p*=0.002), and a poorer quality of life (26.9 ± 18.8 vs. 40.9 ± 26.4, *p* < 0.001) than those in the PD-C cohort. In terms of therapeutic interventions, the PD-MCI group were more likely to be using DBS treatment (OR = 4.72, *p*=0.030) and have a higher LED (765 ± 571 vs. 970 ± 620, *p* < 0.001) than those with normal cognitive function. As such, further analysis of the relationship between impulsivity scores and cognitive status included age at assessment, gender, disease duration, LED, and DBS as covariates.

### 3.3. Impulsivity Scores in PD-C and PD-MCI Groups

Total BIS-11 scores of all patients ranged from 30 to 102, with an interquartile range of 12 points. Patients with PD-MCI, compared to those who were PD-C, scored higher on total impulsivity measures and multiple subscales of impulsivity; however, no differences reached statistical significance (Table 3). BIS-11 subscales were only analysed in corrected comparisons where significance was seen in naïve pairwise comparison.

Specifically, individuals within the PD-MCI group had higher total BIS-11 scores, although not statistically significant (59.5 ± 8.77 vs. 60.2 ± 10.2, *p*=0.318). Second-order attentional subscale findings reflected total BIS-11 scores, with differences between groups remaining minimal with no significance noted (15.3 ± 3.18 vs. 15.4 ± 3.80, *p*=0.704). Within this attentional subscale, first-order attentional subscale scores also saw no significant differences (*p*=0.137), whilst cognitive instability scores were significantly different (*p*=0.019). However, in fully corrected models controlled for confounding factors, cognitive stability was no longer significant in effect (*p*=0.787). Subsequent analysis of second-order nonplanning scores and motor scores indicated no differences in mean scores (23.8 ± 4.81 vs. 23.8 ± 5.67, *p*=0.175; 21.0 ± 3.26 vs. 21.0 ± 2.90, *p*=0.737), mirrored by insignificant differences in first-order subscales (Table 3).

## 4. Discussion

Cognitive dysfunction is an important nonmotor manifestation of PD, which increases in frequency and severity as the disease progresses, and can range from mild impairment in one or more cognitive domains, to outright dementia. People with PD are twice as likely to develop MCI and six times more likely to become demented when compared to age-matched controls [25]. Furthermore, the onset of PD-MCI has been recognized as a predictive factor for other debilitating symptoms such as sleep problems, depression, hallucinations, and ICDs [26]. It has been suggested that cognitive impairment may be involved in the development of ICDs [15, 16, 27], and it has been proposed that the two disorders may share a common underlying neurobiological substrate [8].

While it has previously been suggested that there may be a link between cognitive impairment and trait impulsivity, which is thought to underlie the development of ICDs, we were unable to demonstrate such an association in the present study using the ACE-R cognitive screening protocol. We did not find any significant differences in attentional, nonplanning, or motor impulsivity BIS-11 measures between a PD-MCI group and a cognitively normal PD group (PD-C), when controlling for other confounding factors. The findings of this study therefore suggest that the presence of PD-MCI in PwP does not align with higher levels of subclinical impulsivity, nor is there evidence for an inverse relationship between impaired cognition and impulsivity, or any indication of a change in any specific impulsivity traits in the cognitively impaired PD group. However, the study did not investigate the influence of domain-specific MCI, which may relate to impulsivity subscales.

Contention exists in the literature surrounding the relationship between cognition and ICDs, with some studies in PD cohorts with diagnosed ICDs reporting no differences in cognitive abilities and others reporting a better level of functioning in some tasks assessing cognition [17, 28]. Moreover, conflicting conclusions have been reached in regard to which specific cognitive functions relate to ICDs. In the current study, the PD-MCI group performed significantly lower in all cognitive domains, and it is possible that impairments in some of these domains could lead to higher impulsivity, whilst impairments in other domains could mitigate other aspects of trait impulsivity. The failure to find any such associations in this study may reflect limitations of the BIS-11 self-reporting scale, and further studies employing more sensitive instruments for trait impulsivity and more comprehensive cognitive testing protocols would therefore be worthwhile to explore this possibility further.

No study has yet demonstrated a relationship between impulsivity and cognition in a cohort of PwP, though an association has been established in other populations. The BIS-11 scale quantifies an individual's perception of various behaviors, thoughts, and actions that are associated with impulsivity [20]. It has been suggested that individuals with lower cognitive abilities may be less likely to comprehend the consequences of reporting their impulsive events, and are therefore more likely to disclose impulse-related behaviors and feelings, and to self-report high impulsivity. This phenomenon has been reported in prison inmates [29], and in children with learning difficulties [30] and ADHD [13]. Alternatively, many individuals with higher cognitive abilities are thought to under-report impulsive behaviors due to embarrassment, or because they are fearful of potential consequences in disclosing impulsive behaviors. The extent to which these potential limitations of the self-reported BIS-11 scale may apply to the PD patient cohort is uncertain, although it is possible that individuals who have MCI may be less able to objectively identify problematic behaviors, and that participants in a research study may have different attitudes to reporting impulsive traits.

## 5. Limitations

A number of other limitations warrant consideration when interpreting the findings of the present study. Firstly, the self-report nature of the BIS-11 assessment is likely to introduce a degree of variability and bias in responses. As patients may be reluctant to report impulsive tendencies, this may limit the reliability of the results of the study. While more objective behavioral measures to assess situational “state” impulsivity and to screen for specific personality traits were not employed in this study, it is important that these aspects be addressed in future studies. In regard to cognition, the PD-C and PD-MCI groupings were established using predefined cut-off scores for global MCI, and the study did not investigate the influence of domain-specific MCI, which may relate to impulsivity subscales, or relation between executive function and nonplanning impulsivity. Furthermore, possible overlap exists between certain items in the BIS-11 and ACE-R scales: e.g., the “attentional impulsiveness” subscale in the BIS-11 and the “attention and orientation” domain in the ACE-R. Moreover, the cross-sectional nature of the study precludes any possible conclusions of a causal relationship, or lack thereof, between deficits in specific cognitive domains and impulsiveness.

## 6. Conclusion

The present results have established that subtle shifts in cognitive circuitry, as related to PD-MCI, do not necessarily associate with individual variability in trait impulsivity. Absence of any obvious relationship between the complex multidimensional constructs of impulsivity and cognition suggests that the underlying neurobiology may not relate, at least at a subclinical level. This prompts a need to reconsider the underpinnings of impulsivity in PD as a separate construct to cognition, in order to better characterize and treat symptoms experienced by patients.

## Figures and Tables

**Table 1:** Differences in ACE-R subdomain scores in the PD-C and PD-MCI groups.

	All subjects (*n* = 302)	PD-C (*n* = 189)	PD-MCI (*n* = 113)	Arm comparison
ACE-R total	88.02 (11.3)	94.4 (3.09)	77.4 (12.1)	*t* = 14.6 (*p* < 0.001)
Attention and orientation	17.3 (1.56)	17.9 (0.364)	16.4 (2.18)	*t* = 10.4 (*p* < 0.001)
Memory	21.8 (4.63)	24.3 (1.79)	17.6 (4.88)	*t* = 12.3 (*p* < 0.001)
Fluency	9.23 (3.71)	11.3 (2.28)	5.79 (3.04)	*t* = 12.2 (*p* < 0.001)
Language	24.8 (1.99)	25.4 (1.02)	23.9 (2.75)	*t* = 5.85 (*p* < 0.001)
Visuospatial	14.7 (2.44)	15.5 (1.00)	13.5 (3.42)	*t* = 5.84 (*p* < 0.001)

**Table 2:** Clinical and demographic characteristics of cohort, when grouped by cognitive status.

	All subjects (*n* = 302)	PD-C (*n* = 189)	PD-MCI (*n* = 113)	Arm comparison
Age of assessment (years)	64.4 (9.20)	63.2 (9.48)	66.4 (8.51)	*t* = −2.95 (*p*=0.003)
Gender: male (%)	186 (61.6)	108 (57.1)	78 (69.0)	OR = 4.28 (*p*=0.039)
Disease duration (years)	8.27 (5.60)	7.39 (5.09)	9.75 (6.10)	*t* = −3.57 (*p* < 0.001)
MDS-UPDRS III score	19.9 (13.1)	17.7 (11.0)	23.6 (15.4)	*t* = −3.17 (*p*=0.002)
Total levodopa equivalent dosage (mg/day)	841 (597)	765 (571)	970 (620)	*t* = −3.046 (*p* < 0.001)
Deep brain stimulation treatment (%)	32 (11.0)	14 (4.8)	18 (15.9)	OR = 4.72 (*p*=0.030)
Parkinson's Disease Questionnaire (PDQ-39)	32.5 (23.1)	26.9 (18.8)	40.9 (26.4)	*t* = −4.785 (*p* < 0.001)

**Table 3:** Total BIS-11 and first- and second-order scores in the overall cohort and PD-C and PD-MCI groups.

	All subjects (*n* = 302)	PD-C (*n* = 189)	PD-MCI (*n* = 113)	Naïve pairwise comparison	Covariate corrected comparison
BIS-11 total score	59.7 (9.33)	59.5 (8.77)	60.2 (10.2)	*p*=0.318	NS
Second-order attentional	15.3 (3.42)	15.3 (3.18)	15.4 (3.80)	*p*=0.704	NS
First-order attentional	10.1 (2.66)	9.96 (2.53)	10.4 (2.56)	*p*=0.137	NS
First-order cognitive instability	5.21 (1.58)	5.34 (1.49)	4.98 (1.70)	*p*=0.019	*p*=0.787
Second-order nonplanning	23.4 (5.15)	23.8 (4.81)	23.8 (5.67)	*p*=0.175	NS
First-order self-control	12.07 (3.56)	12.0 (3.29)	12.2 (3.73)	*p*=0.503	NS
First-order cognitive complexity	11.3 (2.58)	11.1 (2.47)	11.57 (2.74)	*p*=0.157	NS
Second-order motor	21.0 (3.51)	21.1 (3.26)	21.0 (2.90)	*p*=0.737	NS
First-order motor	13.6 (2.98)	13.7 (2.77)	13.5 (3.33)	*p*=0.559	NS
First-order perseverance	7.46 (1.71)	7.43 (1.63)	7.50 (1.85)	*p*=0.734	NS

^*∗*^NS = not significant.

## Data Availability

The data that support the findings of this study are available from the corresponding author upon reasonable request.
